# Implementation of Core Entrustable Professional Activities in the Internal Medicine Clerkship: A Psychometric Study

**DOI:** 10.1007/s11606-026-10210-2

**Published:** 2026-01-29

**Authors:** Julia Lister, Shahidali Jaffer, Rachel Stork Poeppelman, Kathleen Lane, Karen Jankowski, Kelsey Angell, Claudio Violato

**Affiliations:** 1 Division of Hospital Medicine, Department of Medicine, University of Minnesota Medical School, Minneapolis, MN USA; 2 Division of Pediatric Critical Care Medicine, Department of Pediatrics, University of Minnesota Medical School, Minneapolis, MN USA; 3 Department of Family Medicine and Community Health, University of Minnesota Medical School, Minneapolis, MN USA; 4 Department of Medicine, University of Minnesota Medical School, Minneapolis, MN USA; 5 University of Minnesota Medical School, Minneapolis, MN USA

**Keywords:** Entrustable professional activities, Competency-based medical education, Internal Medicine clerkship, Workplace-based assessment

## Abstract

**Background:**

Entrustable professional activities (EPAs) have been proposed as a holistic approach to competency-based assessment. The 13 Core EPAs for Entering Residency (CEPAER) are essential tasks that a medical student should be trusted to perform with indirect supervision upon entering residency, based on demonstrated competence.

**Objective:**

To study the validity and reliability of workplace-based assessments of the 13 core EPAs as measurements of medical student performance and growth over the Internal Medicine (IM) clerkship.

**Design:**

Correlational-based population study.

**Participants:**

A total of 398 third-year medical students at the University of Minnesota Medical School participated. Students were enrolled in a required 8-week IM clerkship during the 2023–2024 and 2024–2025 academic years. A total of 825 assessors provided EPA ratings with a mean number of 12 per assessor; SD = 15.08.

**Main Measures:**

There were 10,034 EPA-based assessments collected (mean per student = 25; SD = 6.1). The most frequently assessed EPAs were EPA 6 (Provide an oral presentation of a clinical encounter; *n* = 1866; mean per student = 4.69), EPA 5 (Document a clinical encounter in the patient record; *n* = 1662; mean per student = 4.18), and EPA 2 (Recommend and interpret common diagnostic and screening tests; *n* = 1421; mean per student = 3.57).

**Key Results:**

Regression analyses indicated statistically significant growth in entrustment scores for EPAs 1, 2, 3, 5, 6, 8, 10, and 12. Generalizability analysis showed that to achieve adequate reliability (Ep2 ≥ 0.80), at least 5 assessments were required to be conducted by 5 raters.

**Conclusion:**

EPAs represent a valid and reliable measure for medical student growth during the IM clerkship, particularly for EPAs 1, 2, 3, 5, 6, 8, and 10.

## INTRODUCTION

Competency-based medical education (CBME) provides a framework that is oriented to outcomes and abilities of medical graduates, and is organized around competencies, which are applications of knowledge, skills, and behaviors in a clinical setting. The central idea is that every graduate can practice medicine at an acceptable threshold level, focusing on the learner’s competence rather than content delivery or the time required to complete educational activities.^1–3^ However, determining competence in a clinical setting can be challenging, as it requires reliability and validity in how learners are evaluated despite variable contexts, preceptors, and patient cases.

Entrustable professional activities (EPAs) were proposed as a holistic approach to competency assessment.^4^ In 2014, the Association of American Medical Colleges (AAMC) introduced 13 Core Entrustable Professional Activities for Entering Residency (CEPAER), which are essential tasks of medical practice that a medical student should be trusted to perform with indirect supervision upon entering residency.^5,6^ After the CEPAER were introduced, a nationwide pilot study examined the feasibility of implementing an EPA assessment framework. It showed alignment of EPAs with medical school curricula, but due to wide variability in data regarding entrustment, or students’ ability to complete a certain task with indirect supervision (i.e. at the level of an intern), none of the participating schools felt ready to use EPA-based decisions for promotion or graduation.^7^ This work highlights the need for additional study to understand how EPAs can be implemented in undergraduate medical education.

The University of Minnesota Medical School (UMMS) is one of the institutions involved in another CBME pilot project sponsored by the AAMC, Education in Pediatrics across the Continuum (EPAC), and has been broadly incorporating EPAs into clerkships since 2021. A study within EPAC demonstrated good evidence of validity for the use of EPAs in a longitudinal integrated clerkship.^8^ This was followed by a more comprehensive psychometric analysis examining over 32,000 EPA assessments from UMMS students across all of the third-year clinical rotations, which found that EPAs produced reliable and valid data about students’ growth and performance over time.^9^

In this study, we provide additional validity and reliability data for EPAs on the Internal Medicine (IM) clerkship at UMMS, a single institution, where students rotate at ten different clinical sites. The rationale behind focusing on EPAs on the IM clerkship is multiple. Previous evidence analyzing the use of workplace-based assessments (WBAs) on Pediatrics, Psychiatry, and Surgery electives showed that WBAs are effective in tracking the development of specialty-specific skills.^10–12^ Additionally, while medical schools across the country are incorporating EPA assessments into the IM clerkship, they are doing so without confirmation regarding the validity and reliability of EPAs in that context.^13^ Finally, current literature on WBAs in the IM clerkship is limited, with one institution exploring the reliability of 5 EPAs and finding that a total of 9–11 WBAs were necessary to achieve adequate reliability.^14^ This study examines the evidence for the validity and reliability of all 13 core EPAs to measure student performance over the IM clerkship.

## METHODS

### Learning Theory and Validity Assessment

Learning curves provide evidence of validity by depicting how students develop competence with respect to individual EPAs over time. Thurstone proposed that the relationship between the duration of practice or experience and learning is defined by a negative exponential function: specifically, gains in learning are initially steep, but then plateau as knowledge and skill acquisition occur.^15^ Learning curves can be used to analyze assessments at both group and individual levels. At the group level, they illustrate the average length of time it takes learners to attain an acceptable level of competence in a specific skill. At the individual level, they show the developmental trajectory for each learner. As a result, learning curves can inform decisions regarding (a) the average duration of time required of learners to reach a predefined competency level, (b) how to focus educational resources to achieve individual and program goals, and (c) how to enhance curricula, teaching, assessments, and feedback to better suit learner needs.^16,17^

Generalizability theory (G-theory) is another approach to collecting validity evidence. G-theory provides a framework for conceptualizing, investigating, and designing reliable measurements and observations.^18^ G-theory is an extension of classical test theory and can be applied when multiple assessors produce score variability. In G-theory, observations are generalized to a universe of observations from which they were sampled, specifying a universe of conditions over which to generalize (such as EPAs, and occasions of assessment).^19^ G-studies can help us identify what contributes to the actual score on an EPA assessment. Finally, Decision studies (D-studies) can also be used to analyze the reliability of EPA data to determine the optimal number of assessors and assessments required.

### Data Collection and Procedures

Students were assessed during their IM clerkship on the 13 CEPAER described in Table 1. They were required to obtain 3 EPA assessments per week during the 8-week clerkship (24 total), with at least one being EPA 1. EPAs were performed by clinical supervisors including faculty, chief residents, and residents.

The EPA assessment form consists of a rating on a 5-point scale and two narrative comment fields—strengths and areas for improvement. The entrustment scale anchors are (1) observation only: “I [resident, faculty] did it. The student observed,” (2) direct supervision: “We did it together,” (3) direct supervision: “I supervised and helped the student from time to time,” (4) indirect supervision: “The student did it. I double-checked ALL elements,” and (5) indirect supervision: “The student did it. I double-checked KEY elements.”

After a student is observed performing an EPA, they access the EPA rating form electronically through a secure Qualtrics survey. The clinical supervisor provides the entrustment rating and narrative feedback and submits the survey.

### Data Analysis

The units of analysis for this study are assessments completed on each of the 13 EPAs. The entrustment rating 5-point scale is ordinal, similar to the Likert scale. An analytic approach employed for these scales is based on the assumption that they are interval or “quasi-interval.” Numerous Monte Carlo studies support the use of these parametric approaches in the field of ordinal analysis. The resulting estimate of the population parameter is almost always the best in that it is closest to the true value. Student ratings are depicted as curves describing their progress on the 5-point entrustment scale over time; regression models were employed to fit the theoretical curves to the actual data.

### Regression Analyses

To describe the growth of students over the course of the IM clerkship, we conducted regression analyses hypothesized as being representative of the relationship between the time variable (weeks) and students’ average ratings on the entrustment scale. We aimed to determine if the growth curve of each of the EPAs would demonstrate a curvilinear structure (e.g., negative exponential), as hypothesized by Thurstone’s learning curve theory. Thus, we examined curvilinear relationships at the group level, employing random effects analysis.

### Generalizability Analysis: D-Study

We employed a two-facet (occasion[F1] × assessor[F2]) nested design [Student*(F1: F2)] to derive G-coefficients for a D-study for the reliability of the EPA Assessments. A sample (*n* = 72) that had all been assessed on 6 occasions on EPA 1 was selected for the G-theory analysis.

### Ethical Approval

This study was determined to be exempt from review by the University of Minnesota–Twin Cities Institutional Review Board.

## Results

### Participants

A total of 398 UMMS medical students participated. All students were enrolled in an 8-week IM clerkship, taking place at 10 clinical sites across the state from 2023 to 2025. A total of 825 assessors provided at least one EPA rating: 528 (64%) residents and 297 (36%) faculty members. Ratings from all 13 core EPAs were included. The mean number of ratings per assessor was 12.2 (SD = 15.08; range = 1 to 438).

### Descriptive Statistics

Ten thousand thirty-four EPA-based assessments were completed for the 398 students (mean number of assessments per student = 25; SD = 6.1). The most frequently assessed EPAs were EPA 6 (Provide an oral presentation; *n* = 1866 ratings; mean = 4.69), EPA 5 (Document a clinical encounter; *n* = 1662 ratings; mean = 4.18), and EPA 2 (Develop and prioritize a differential diagnosis; *n* = 1421 ratings; mean = 3.57).

By contrast, lower-frequency EPAs included EPA 13 (Culture of safety and improvement; *n* = 109 ratings; mean = 0.27), EPA 11 (Obtain informed consent; *n* = 149 ratings; mean = 0.37), EPA 10 (Recognize a patient requiring urgent or emergent care; *n* = 160 ratings; mean = 0.40), and EPA 8 (Give or receive a patient handover; *n* = 168 ratings; mean = 0.42). Table 1 summarizes the descriptive statistics for each EPA, as well as the results of the regression analyses, which include the slope of the growth curves (*β*_0_) and the goodness-of-fit (R^2^).

**Table 1:** IM Descriptive Statistics for Entrustable Professional Activities (EPAs), Regression Goodness-of-Fit (R^2^), and Slope (*β*_0_) of the Growth Curves

Entrustable professional activities	Total # of ratings	Mean # ratings/student	R^2a^	***β***_0_ (Slope^b^)
EPA 1: Gather a history and perform a physical examination	1320	3.32	0.91	.085*
EPA 2: Develop and prioritize a differential diagnosis following a clinical encounter	1421	3.57	0.99	.125**
EPA 3: Recommend and interpret common diagnostic and screening tests	1127	2.83	0.99	.126**
EPA 4: Enter and discuss orders and prescriptions	684	1.72	0.41	.038
EPA 5: Document a clinical encounter in the patient record	1662	4.18	0.82	.143**
EPA 6: Provide an oral presentation of a clinical encounter	1866	4.69	0.93	.096*
EPA 7: Form clinical questions and retrieve evidence to advance patient care	632	1.59	0.48	.036
EPA 8: Give or receive a patient handover to transition care responsibility	168	.42	0.96	.157**
EPA 9: Collaborate as a member of an interprofessional team	585	1.47	0.52	.033
EPA 10: Recognize a patient requiring urgent or emergent care and initiate evaluation	160	.40	0.94	.162**
EPA 11: Obtain informed consent for tests and/or procedures	149	.37	0.20	.001
EPA 12: Perform general procedures of a physician	151	.38	0.41	.113*
EPA 13: Identify system failures and contribute to a culture of safety and improvement	109	.27	0.53	.044*

^a^R^2^ is a fit index for the regression line to the raw data—0 to 1; 0 = no fit, 1 = perfect fit

^b^The slope (*β*_0_) of the negative exponential (*e*) EPA growth curves of the form, *Y* = *β*_0_*X*^*e*^ + *c*. The larger the value *β*_0_, the steeper the learning curve (Fig. 1)

^*^<.01; **<.001

### Growth (Learning Curves)

**Figure 1:**
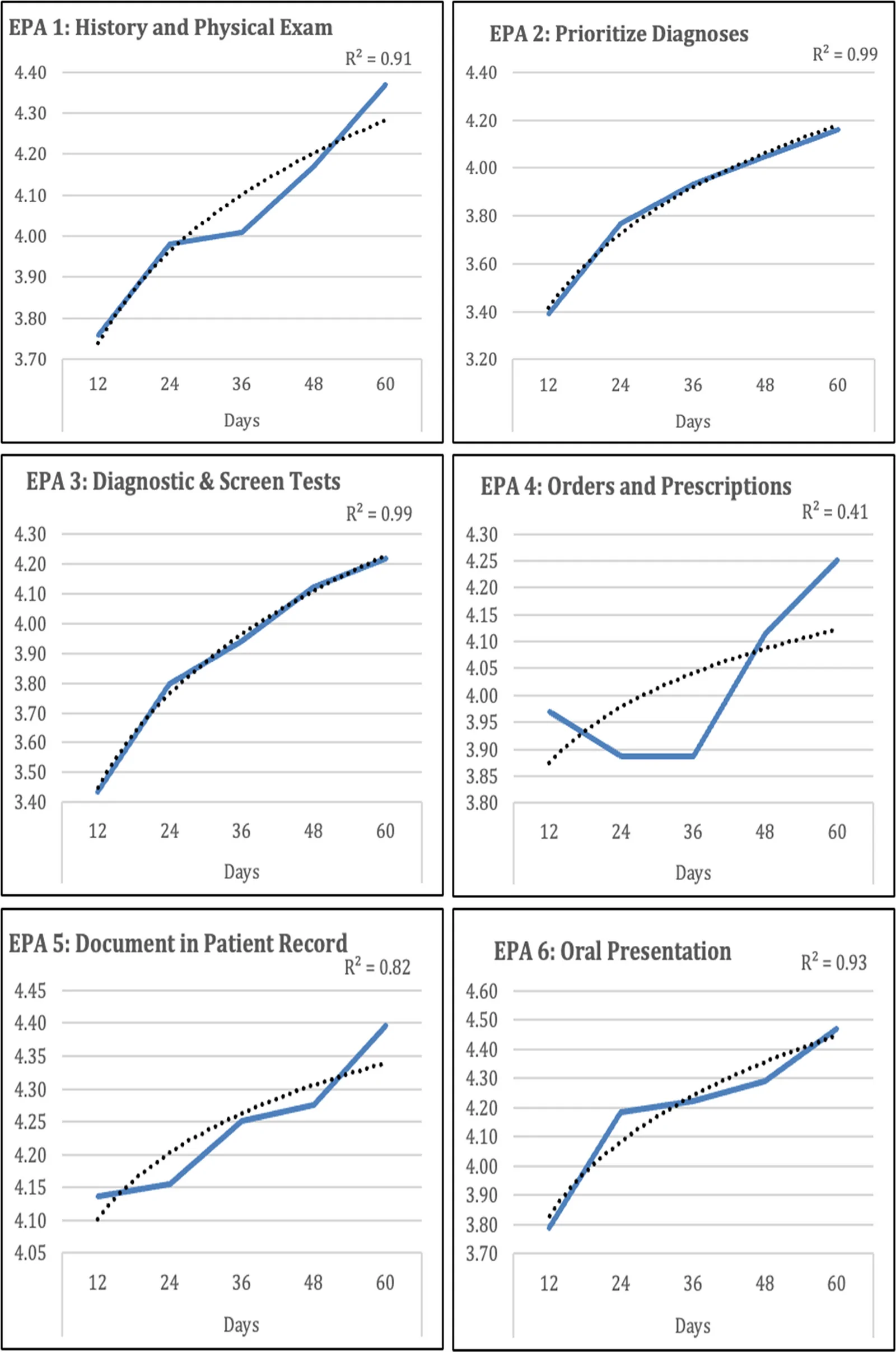

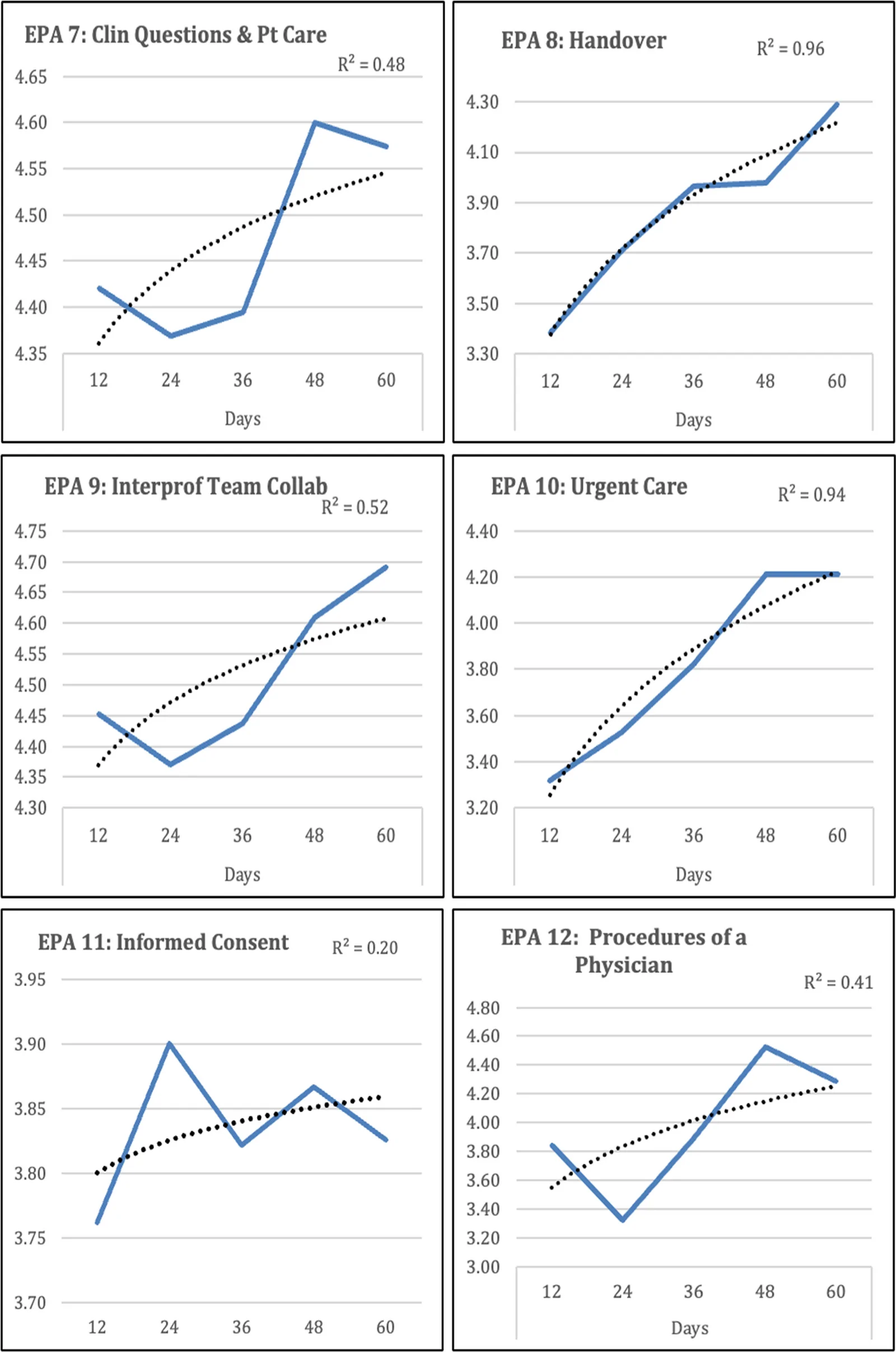

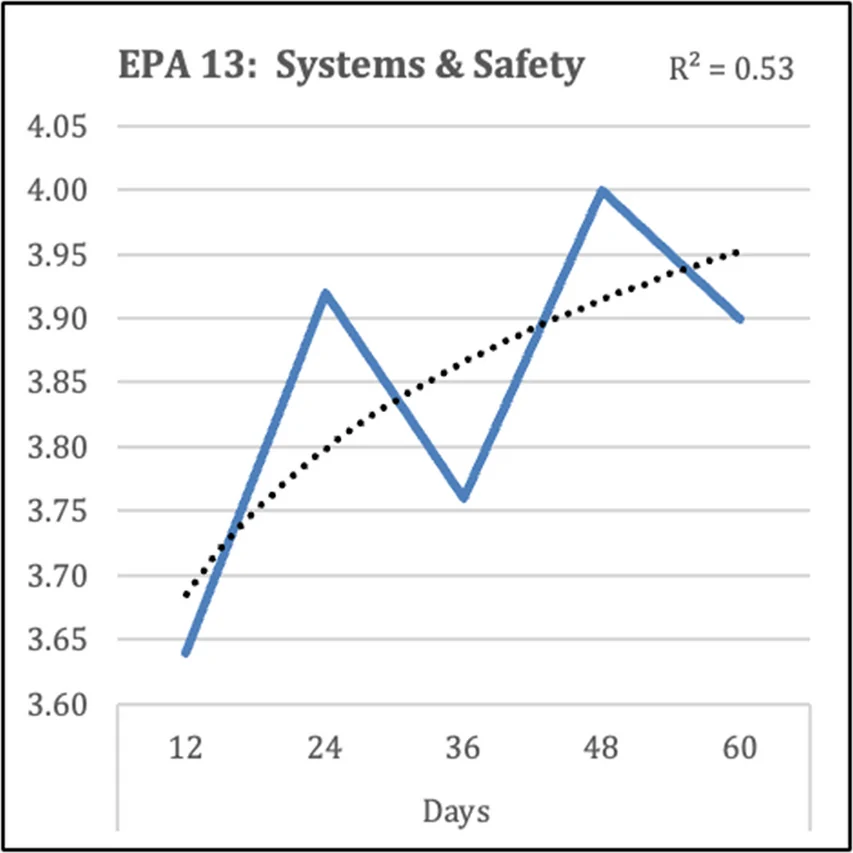
Entrustable professional activities (EPA) growth curves for the Internal Medicine clerkship. EPA ratings are on the Y axis. The solid line is raw data while the dotted line is the fitted curve

Figure 1 displays learning trajectories for each EPA across the 8-week Internal Medicine clerkship. Solid lines represent raw student ratings over time, while dotted lines depict fitted negative exponential curves based on regression modeling. To analyze the growth curves, the 8-week (60 day) IM rotation was binned into equal time units (12 days) in order to collect sufficient data at each time point to plot means for the raw data and fit the theoretical non-linear regression line with the goodness-of-fit index.

EPA data are generally collected evenly over the time points. The percentages ranged from a low of 15% to a high of 25% and did not always occur at time 1 (12 days) or time 5 (60 days). This distribution was true even for high data EPAs (e.g. EPA 6: *n* = 1866) and low data EPAs (e.g., EPA 12: *n* = 151). For EPA 1, for example, the *n* and percent distribution over the 5-time points were 232 (17.5%), 279 (21.1%), 302 (22.9%), 273 (20.7%), and 234 (17.8%); total = 1320.

The regression analyses indicated statistically significant growth (*p* < 0.01; 0.001) in entrustment scores for EPAs 1, 2, 3, 5, 6, 8, 10, and 12. EPAs 4, 7, 9, 11, and 13 did not show statistically significant growth, as reflected by lower R^2^ values and smaller or non-significant *β*_0_ slopes.

By the end of the clerkship, student performance on several EPAs approached or exceeded a score of 4 on the entrustment scale, indicating readiness for indirect supervision.

### Reliability Analysis (G-Theory)

**Figure 2:**
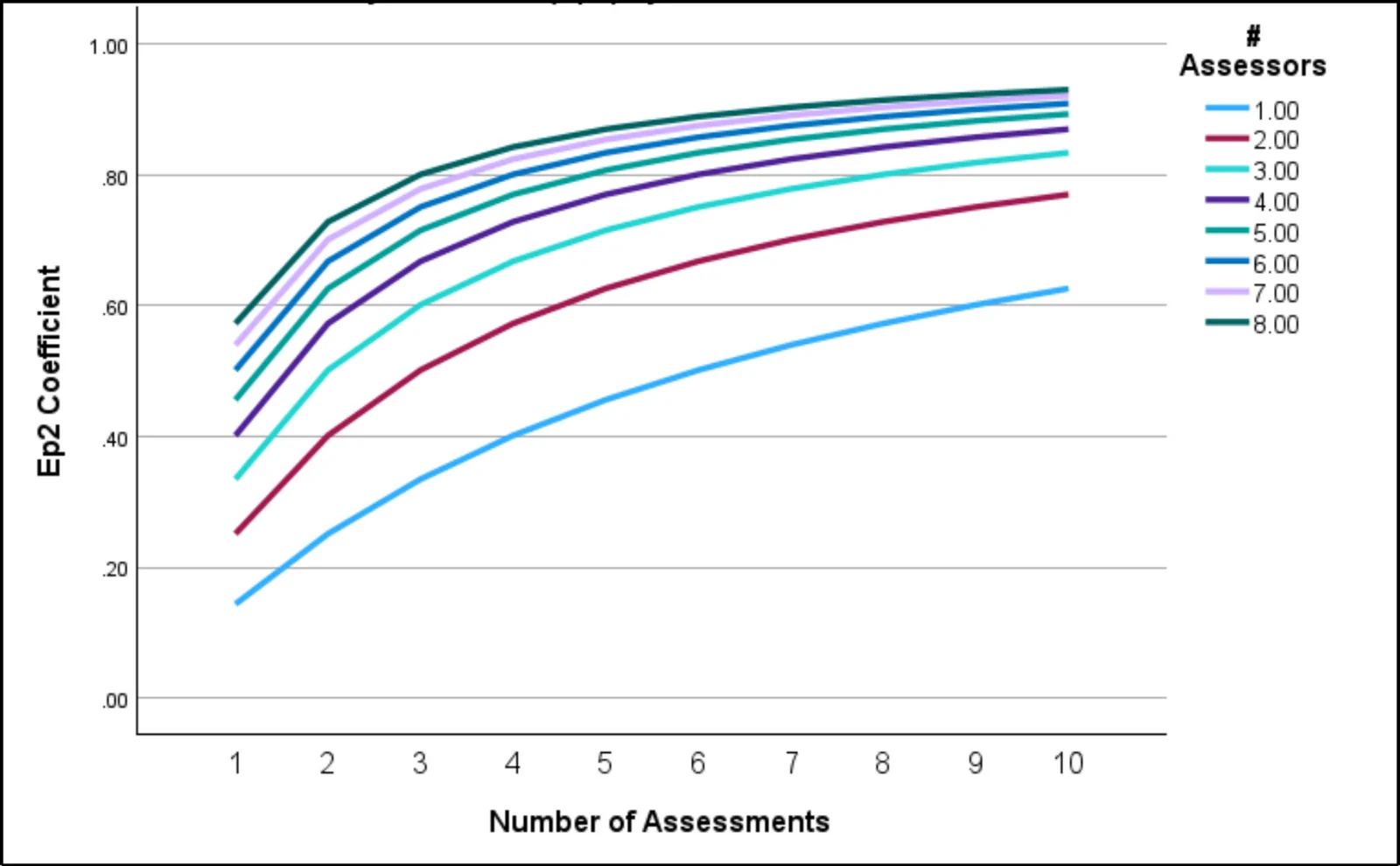
D-study showing generalizability coefficient (Ep2) by number of assessors and number of assessments. Adequate reliability is defined as an Ep2 > 0.80 which in our study required at least five assessments by 5 assessors

We adapted SPSS algorithms for these analyses based on the work of Mushquash and O’Connor,^20^ using a two-facet (occasion[F1] × assessor[F2]) nested design [Student*(F1: F2)] to derive G-coefficients reliability calculations with a sample (*n* = 72) that had all been assessed on at least 6 occasions on EPA 1. We chose EPA 1 for this analysis because it had a large number of assessments and assessors, allowing for robust data analysis on reliability as we had found in a previous study.^8^ To achieve adequate reliability (Ep2 > 0.80), 5 assessments were required to be conducted by 5 raters (Fig. 2).

## DISCUSSION

Our data support the reliability and validity of EPA assessments in tracking student growth in clinical skills during the IM clerkship. This is most clearly seen in the statistically significant growth demonstrated in EPAs 1, 2, 3, 5, 6, 8, and 10. For these EPAs, students began at a level of needing direct supervision (a rating of 1–3 on the 5-point scale) and progressed to a level of indirect supervision (rating of 4 or 5) by the end of their rotation. Critically, these EPAs also represent core skills that align with the expectations of students on the IM clerkship.

Although EPA 12 (procedures) also showed statistically significant growth reflected by the slope (*β*_0_) of the growth curve, the low R^2^ value suggests it does not follow the expected learning trajectory, and therefore, we did not consider it to be good evidence for validity of EPA 12. This is not surprising, as procedures are generally not sought out or expected of students during the IM clerkship, and thus we would not expect to see significant growth in procedural skills on this rotation. Another EP A that did not follow the expected learning growth curve was EPA 4 (enter and discuss orders). While this is a skill we expect to see growth in during the IM clerkship, we believe several factors contribute to this observed pattern. First, there are many interpretations of what EPA 4 signifies among assessors, with many feeling that this skill is more suitable for a sub-intern. Additionally, while some assessors evaluate students on EPA 4 based on a proposed treatment plan for a patient, others focus on students’ particular ability to navigate the electronic medical record to place orders. For clerkship directors, this may be both an opportunity for faculty development on EPA 4 and a fruitful area of curriculum development for clerkship students.

For some EPAs that did not show good fit, such as EPA 7 (clinical questions) and 9 (interprofessional team), students began their clerkship at a relatively high rating (> 4), limiting the amount of growth observed during their clerkship—though notably, there was still some progression in these skills. Additional EPAs that did not follow the expected growth curves include EPA 11 (informed consent) and 13 (patient safety). For these EPAs, the lack of sufficient assessments limited data quality. Similar to EPA 12, opportunities to practice EPA 11 may be limited in IM, and students typically seek out this EPA on different clerkships. EPA 13, however, was expected to be an essential facet of the IM clerkship and represents an opportunity for further exploration and curriculum development to better incorporate this EPA. Overall, the EPAs that did not fit the expected learning curves in our analysis are neither surprising nor inappropriate, as they represent skills that students acquire during different clerkships or during their sub-internship rotations.

Regarding generalizability, we found that a minimum of 5 evaluations by 5 different assessors was required to provide adequate reliability regarding a student’s level of supervision needed. To our knowledge, the only other study assessing the specific reliability of EPAs on the IM clerkship found that 9–11 assessments were required for reliability across 5 EPAs.^14^ Our study builds on this work by analyzing a larger number of students and assessments and including data on all 13 core EPAs. While our data suggest that valid and reliable assessments in EPA performance for medical students are achievable on the IM clerkship, the specific number of assessors and assessments required can be variable and are likely institution-specific. Broader assessments of EPA reliability have reflected this; for example, Ryan et al. assessed reliability data across multiple institutions and rotations, and found low reliability in WBAs between institutions, with the number of assessments needed ranging from 3 to more than 560.^21^

We believe the reason behind our lower number of assessments needed for reliability is multifactorial. First, as noted previously, our institution has a strong foundation for EPA assessment from the work of EPAC, which was then broadened to include EPAs as a standard form of assessment across all required third-year clerkships. Also, a key part of EPA implementation at UMMS was the development of a group of Assessment and Coaching Experts (ACEs), who are faculty specially trained in the CEPAER and provide one-on-one student coaching in EPA performance as well as education on EPA assessments to attendings and residents. Finally, as a response to feedback, we now use a modified entrustment scale which has been simplified and adjusted over time to increase ease and accuracy of assessments. We believe these efforts have greatly improved assessor and student comfort with EPAs.

Although our results support the overall validity and reliability of EPA assessments on the IM clerkship, we believe that each institution will need to assess its own reliability data to determine the appropriate number of assessments and assessors needed to evaluate student performance in EPAs. It is also important to note that while clerkship-specific EPA data is useful for tracking student growth in the CEPAER, it is not the only metric used at our institution for summative entrustment decisions. Rather, at UMMS we have trained groups of faculty, including ACEs, who form Entrustment Committees (ECs). ECs decide students’ entrustability in the CEPAER based on their individual EPA data across all of the required clerkships, to ensure the student has repeatedly demonstrated entrustability in a variety of contexts over time. Students cannot be entrusted based on the data from a single clerkship.

While encouraging, this study has notable limitations. First, our data is generated from a single institution and a single clerkship. Second, several EPAs had smaller numbers of assessments, which limited our validity analysis. Additionally, while we found statistically significant growth over time for many EPAs in the IM clerkship, the absolute change in some cases is less than 1 point on the rating scale. This may demonstrate improvement in competency across all learners, but might not mean significant progress at the individual level. Finally, with regard to reliability, our results are based on a D-study of only EPA 1, chosen because of the high number of assessments and assessors associated with it. We are extrapolating reliability in EPA 1 to the other EPAs while also acknowledging that each EPA has its own challenges in evaluation of student performance. In addition, the reliability analysis showing the need for 5 assessments by 5 assessors reveals a practical limitation to students demonstrating competence in all 13 EPAs on the IM clerkship, as it would require obtaining a total of 65 WBAs. This highlights the need to prioritize the selection of EPAs based on the best alignment with learning objectives for the rotation. We also recognize that not every institution has trained assessment coaches to provide student and faculty development around EPAs, which limits the generalizability of this study to other medical schools.

## CONCLUSION

Overall, our data provide evidence of the reliability and validity of EPA use on the IM clerkship. Students on this rotation should expect to see growth in EPA 1, 2, 3, 5, 6, 8, and 10. Weaker data for EPAs 4, 7, 9, 11, 12, and 13 reflect that students started the rotation at a level of indirect supervision, had limited access to particular EPAs better observed on other rotations, and provide opportunities for faculty and curriculum development. Generalizability analysis suggests that at least 5 assessments by 5 different assessors are needed for reliability which is a lower number than others have found, and which we believe reflects our institutional comfort with the use of EPAs.

## Data Availability

The data included in this article contains protected student information and is not available to be shared.
